# Possible transport evidence for three-dimensional topological superconductivity in doped *β*-PdBi_2_

**DOI:** 10.1038/s41598-019-48906-7

**Published:** 2019-08-29

**Authors:** Ayo Kolapo, Tingxin Li, Pavan Hosur, John H. Miller

**Affiliations:** 1 Texas Center for Superconductivity and Department of Physics, University of Houston, 3201 Cullen Boulevard, Houston, Texas 77204 USA; 2 Department of Physics and Astronomy, Rice University, Houston, Texas 77251 USA

**Keywords:** Materials science, Superconducting properties and materials, Topological matter, Topological insulators

## Abstract

Interest in topological states of matter burgeoned over a decade ago with the theoretical prediction and experimental detection of topological insulators, especially in bulk three-dimensional insulators that can be tuned out of it by doping. Their superconducting counterpart, the fully-gapped three-dimensional time-reversal-invariant topological superconductors, have evaded discovery in bulk *intrinsic superconductors* so far. The recently discovered topological metal *β*-PdBi_2_ is a unique candidate for tunable bulk topological superconductivity because of its intrinsic superconductivity and spin-orbit-coupling. In this work, we provide experimental transport signatures consistent with fully-gapped 3D time-reversal-invariant topological superconductivity in K-doped *β*-PdBi_2_. In particular, we find signatures of odd-parity bulk superconductivity via upper-critical field and magnetization measurements— odd-parity pairing can be argued, given the band structure of *β*-PdBi_2_, to result in 3D topological superconductivity. In addition, Andreev spectroscopy reveals surface states protected by time-reversal symmetry which might be possible evidence of Majorana surface states (Majorana cone). Moreover, we find that the undoped bulk system is a trivial superconductor. Thus, we discover *β*-PdBi_2_ as a unique bulk material that, on doping, can potentially undergo an unprecedented topological quantum phase transition in the superconducting state.

## Introduction

According to the traditional Landau-Ginzburg paradigm, states of matter are defined by the symmetries broken in thermal equilibrium that are preserved by the underlying Hamiltonian, and phase transitions acquire universal features that only depend on the symmetries involved and the spatial dimension. However, this definition proves inadequate for topological phases, in which the ground state wavefunction of the bulk system is characterized by a global, topological quantum number which distinguishes it from a conventional phase with the same symmetries^1–3^. Naturally, critical points separating these phases fall outside the traditional paradigm as well. The most striking consequence of the non-trivial bulk topology is the presence of robust surface states where the bulk terminates.

One of the most celebrated topological phases in condensed matter systems is the time-reversal symmetric strong topological insulator (TI) in three dimensions, which is characterized by a ${{\bf{Z}}}_{2}$ topological invariant *ν* = odd/even^4,5^. The surface manifestation of the bulk topology in this phase is the presence of an odd number of pseudo-relativistic, helical surface states (Dirac Cone) that are robust against non-magnetic perturbations. Numerous materials have been predicted to be in this phase, and many of them have been experimentally confirmed. Additionally, several TIs can be tuned into trivial insulators with doping, thus allowing experimental access to the quantum critical point separating them.

A close cousin of the topological insulator is the time-reversal symmetric topological superconductor (TSC) in 3D (Class 3D III)^2^. Here, the superconducting gap plays the role of the insulating gap of the insulator, the topological invariant is $\nu \in {\bf{Z}}=\mathrm{0,}\,\mathrm{1,}\,2\ldots$, and the surface hosts *ν* helical Majorana fermions (Majorana cone) instead of Dirac fermions (Dirac Cone). The *sufficient* conditions for 3D time-reversal invariant (TRI) topological superconductivity are: *One*, the normal state Fermi surfaces enclose an odd number of time-reversal invariant momenta, *two*, the bulk superconductivity is fully gapped, and *three*, odd-parity^6,7^. Once these conditions are met, the surface states are spanned by robust, helical Majorana surface states. The 2D Majorana surface states also referred to as the Majorana cone (can be regarded as the superconducting analog of the 2D Dirac cone)— and is distinct from the Majorana Zero Mode (MZM). The transport signatures of 2D Majorana surface states are also distinct from that of MZM^8,9^. MZM has long been shown to exist in various 1D and 2D heterostructures of s-wave superconductors and spin-orbit coupled systems including topological insulators^10,11^, and in vortex core of some 2D topological metals^12,13^.

The main materials platform that has been studied experimentally for 3D/bulk topological superconductivity is the prototypical TI Bi_2_Se_3_ doped with Cu^14–16^. Unfortunately, undoped Bi_2_Se_3_ does not display superconductivity at ambient pressures, so a topological-to-trivial superconductor phase transition does not occur in this system. A unique bulk material candidate is the intrinsically superconducting topological metal, *β*-PdBi_2_— a layered, centrosymmetric, tetragonal compound. Spin-angle-resolved photoemission spectroscopy (spin-ARPES) and quasiparticle interference imaging have revealed the presence of spin-polarized topological surface states around *E*_*f*_ in the non-superconducting state^17,18^. Intrinsic spin-orbit-coupling (SOC) and superconductivity robust to different dopants, in addition to a relatively high T_*c*_ compared to other systems, make this material an attractive candidate for realizing tunable bulk topological superconductivity. *β*-PdBi_2_ already fulfills the first and second sufficient conditions, and can potentially fulfill the third condition. Although existing experiments show that the bulk superconductivity in pristine *β*-PdBi_2_ is s-wave^19,20^, because of intrinsic SOC, it can also be odd-parity. It is now well known that in the presence of SOC, electron-phonon interaction can give rise to even- as well as odd-parity superconductivity pairing^21–23^. Studies have shown that the s-wave, even-parity state invariably onsets at a higher T_*c*_, driving the odd-parity state to T = 0^21^. Suppressing the s-wave pairing channel can promote the odd-parity channel^22,23^.

In this work, we investigate the superconducting transport properties of *β*-PdBi_2_ tuned with K dopants. The main findings is that in layered, centrosymmetric topological metals (i.e with strong SOC), doping can be a tuning parameter between even- and odd-parity superconductivity. Since the parity of the bulk superconductivity is tied to the topological classification, doping can, therefore, drive a ‘trivial’ superconductor into a strong topological superconductor in these unique materials. Specifically in this report, we find signatures of unconventional bulk superconductivity in K-doped *β*-PdBi_2_: the upper-critical field exceeds the prediction by the Werthemer-Helfand-Hohenberg (WHH) orbital model for conventional *s*-wave pairing, but is consistent with the prediction for polar *p*-wave pairing. With odd-parity superconductivity in the bulk, helical Majorana fermions are expected to emerge as the 2D topological surface states. As the current ARPES systems do not have enough resolution to directly detect in-gap Majorana states, transport experiments via tunneling or Andreev spectroscopy is still the most direct experimental probe. Our point-contact spectroscopy (PCS) experiment in the Andreev spectroscopy regime shows signatures of helical surface states protected by time-reversal symmetry, consistent with the prediction for time-reversal-invariant 3D topological superconductors. Thus, K-doped *β*-PdBi_2_ is likely to be a 3D bulk topological superconductor and could undergo an unprecedented topological superconducting phase transition between a trivial (undoped) and a topological (doped) superconductor. If there is an intermediate magnetic phase, the TSC-magnetism critical point would be a condensed matter realization of supersymmetry^24^.

## Bulk Superconducting Transport Properties

### Magnetization: Normal state background

**Figure 1:**
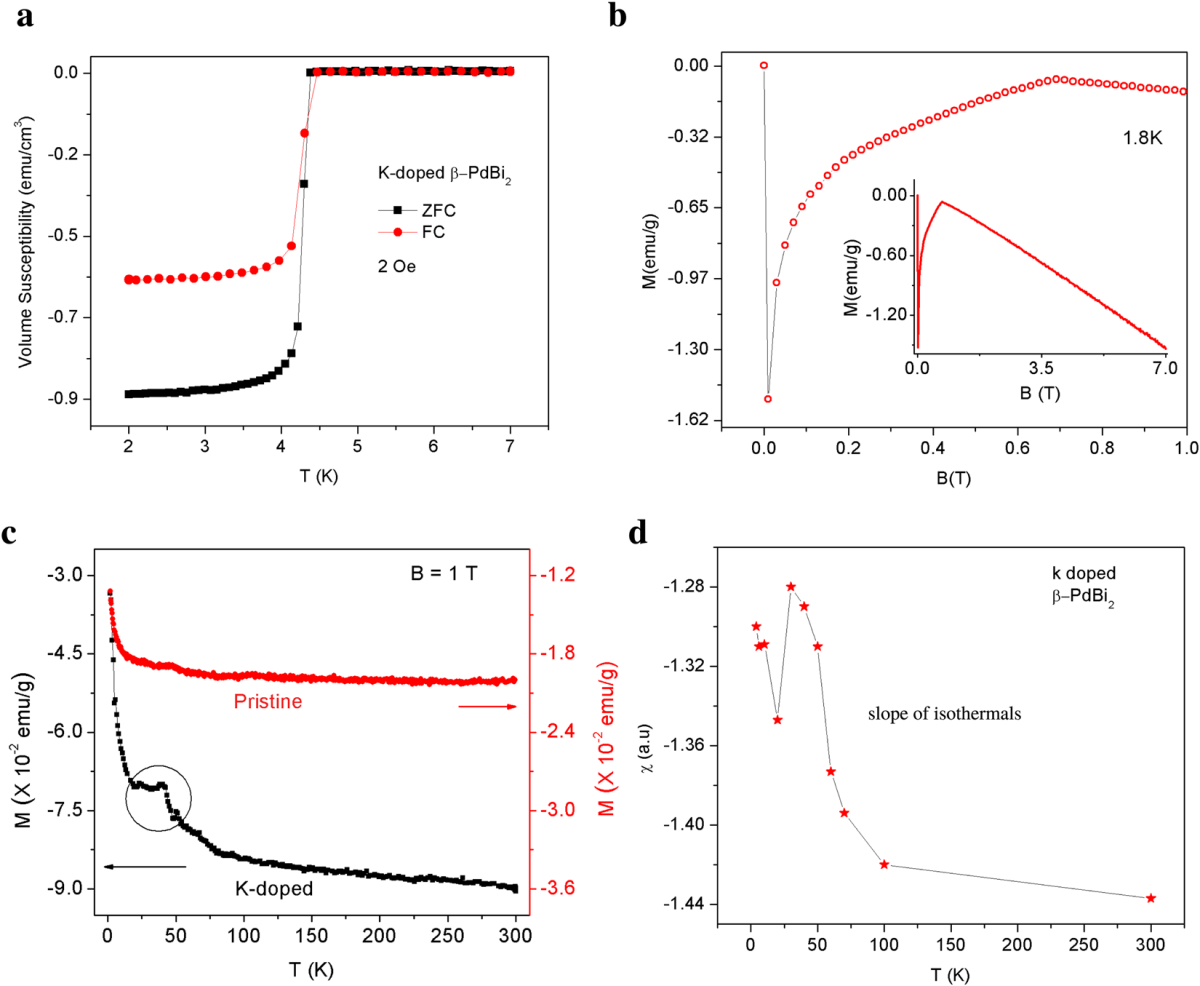
Basic transport properties of K-doped *β*-PdBi_2_. (**a**) Meissner effect in K-doped *β*-PdBi_2_. Zero-field-cooled (ZFC) data reveals bulk superconductivity onset at 4.4 K. (**b**) Magnetization as a function of applied magnetic field (*M*-*B*): plot suggests the upper critical field at 1.8 K is 0.68T. The inset shows that the non-superconducting state is diamagnetic. (**c**) Magnetization of K-doped *β*-PdBi_2_ in the non-superconducting state, showing a hump around 30 K. Such feature is characteristic of spontaneous spin ordering. (**d**) Theintrinsic magnetic susceptibility *χ* = *M*/*B* is calculated from the slope of the plots of isotherms of the magnetization vs magnetic field at several temperatures. The results reveal that the magnetic ordering arising close to low temperatures is intrinsic to the sample.

We begin by studying the basic normal state background from which the superconductivity arises in the pristine (onset T_*c*_ = 5.3 K) and potassium doped (0.3%) *β*-PdBi_2_ (T_*c*_ = 4.4 K). Above the superconducting transition temperature, evidence of topological Dirac surface states is found in the magnetoresistance via features of weak antilocalization^25^. In Fig. 1 we study the magnetization of the superconducting and normal state background. To observe the Meissner effect, we cooled the sample from room temperature down to 1.8 K and applied a small magnetic field ~2 Oe (Zero-Field Cooled, ZFC). The Meissner effect observed in the ZFC measurement on the K-doped sample is displayed in Fig. 1a. From this information, we derived the superconducting volume fraction, estimating that about 93% of the sample volume is superconducting. The magnetization vs magnetic field at 1.8 K displayed in Fig. 1b, shows that the parent (non-superconducting state) of the system is diamagnetic, as expected for topological insulators.

Shown in Fig. 1c is the temperature dependence of the zero-field-cooled magnetization of the doped sample. A magnetic field of 1T, higher than the upper-critical field, is applied along the c-axis. The sample is diamagnetic, except that at around 30 K, the magnetization displays an anomalous hump. Such a feature— concave hump in magnetization and susceptibility versus temperature— is characteristics of spontaneous spin ordering. To confirm that this hump is intrinsic to the sample^26^, we performed isothermal magnetization *M*(*B*) measurements. The intrinsic magnetic susceptibility *χ* is then extracted at different temperatures from the slope of the isotherms: *M*(*B*) ∝ *χB*. The hump in *χ*(*T*) is found to be intrinsic to the sample as shown in Fig. 1d. We discuss the possible origin of this feature in the discussion section. This magnetic excitation in the vicinity of superconductivity can suppress s-wave pairing in favor of spin-triplet paring.

### Magnetization: vortex state

**Figure 2:**
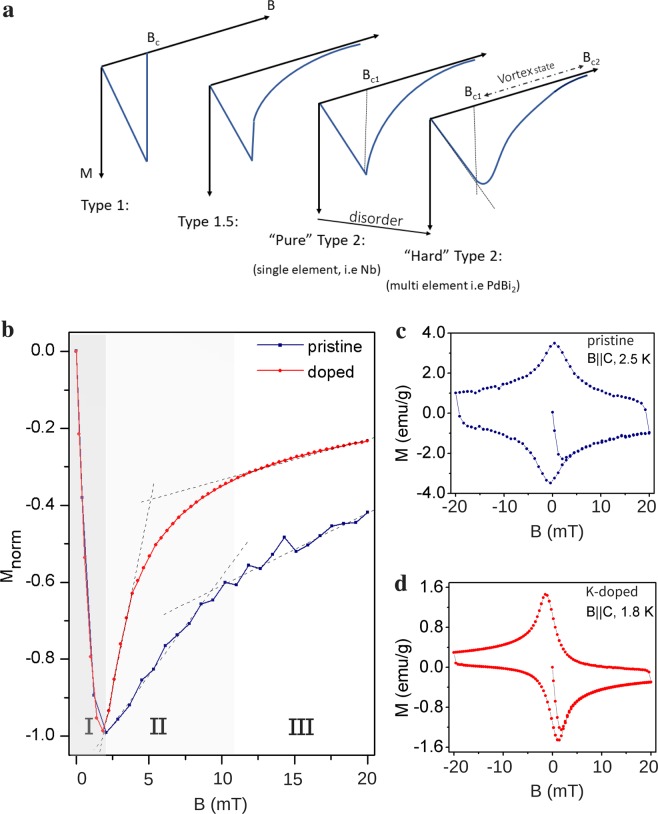
Vortex State. (**a**) *B*_*c*1_ < *B* < *B*_*c*2_ is the vortex state: flux vortices start to penetrate the superconductor at *B*_*c*1_ and form a vortex lattice. Irreversibility of the magnetization loop is due to flux pinning effects. At the irreversibility field, the vortex lattice melts, restoring the reversibility. Doping increases the number of pinning sites and consequently, irreversibility. However, a spin-triplet superconductor does not follow this conventional behaviour; their vortex state is mostly ‘liquid’, resulting in poor flux pinning and reversibility. This behaviour is dubbed *Type 1.5*^27^. (**b**–**d**) Comparison of the normalized magnetization along the *B*||*c* plane of both the undoped and doped sample, at 2.5 K and 1.8 K respectively. Beyond *B*_*c*1_ of K-doped *β*-PdBi_2_ the magnetization shows an anomalous rate of increase in magnitude which is at odds with the conventional type-II “alloy” superconductors but consistent with the expectation for spin-triplet pairing.

As a first check for the transport signature of the effect of the magnetic excitation in the vicinity of superconductivity, we study the vortex state. We recall that for Type 2 superconductors the *vortex state*— the intermediate state in which the superconducting state coexist with a ‘lattice’ of vortices created by penetrating magnetic field— is strengthened by doping. Doping creates defects which pin the vortices, increasing the irreversibility field *B*_*hir*_ and reducing the slope of magenetization curves beyond the lower-critical field *B*_*c*1_. In the Fig. 2a, we illustrate the magnetization curves expected for superconductors; for Type 2 superconductors we emphasized the effects of the disorder. “Pure” type 2 superconductors refers to the limit of clean systems in elemental superconductors, while “Hard” superconductors refer to disordered superconductors. We expect K-doped *β*-PdBi_2_ to be relatively disordered compared to the pristine sample, thus exhibiting more of the “hard” Type 2 behaviour.

In Fig. 2b, we compare the magnetization of the pristine crystal with the doped one. Below *B*_*c*1_, labeled region I, diamagnetization occurs at the same rate for both systems; in region II, however, an anomalous behavior of the rate of magnetization is observed. The magnetization of doped sample behaves as if it is a *cleaner* sample in comparison with the pristine system!

The magnetization for a spin-triplet superconductor, as demonstrated for Cu_*x*_Bi_2_Se_3_^27^, or in magnetic superconductors^28^ exhibits the so-called Type 1.5 like behavior (Fig. 2a). Consider the effect of the magnetic field induced by the persistent vortex current on the spins of the spin-triplet pairs. The induced magnetic field polarizes the Cooper pairs and an additional spin magnetization arises. The total magnetic flux in the vortex now consists of the current and spin magnetization contributions— and is quantized. The quantization of magnetic flux causes current inversion in parts of the vortex, favoring their formation just above *B*_*c*1_ by driving an attractive interaction between the vortices. In the study by Das *et al*.^27^, the attractive interaction does *not* occur in s-wave superconductors. This explains the anomalous increase of the magnetization past the *B*_*c*1_. Because the attractive interaction ‘melts’ the vortex lattice, low irreversibility is often observed in the magnetization vs magnetic field loop of Cu_*x*_Bi_2_Se_3_^15^. This proposes that low irreversibility and anomalous magnetization in doped *β*-PdBi_2_, which is absent in the pristine sample, can also be explained by spin-triplet pairing.

### Upper-critical field limiting effect

**Figure 3:**
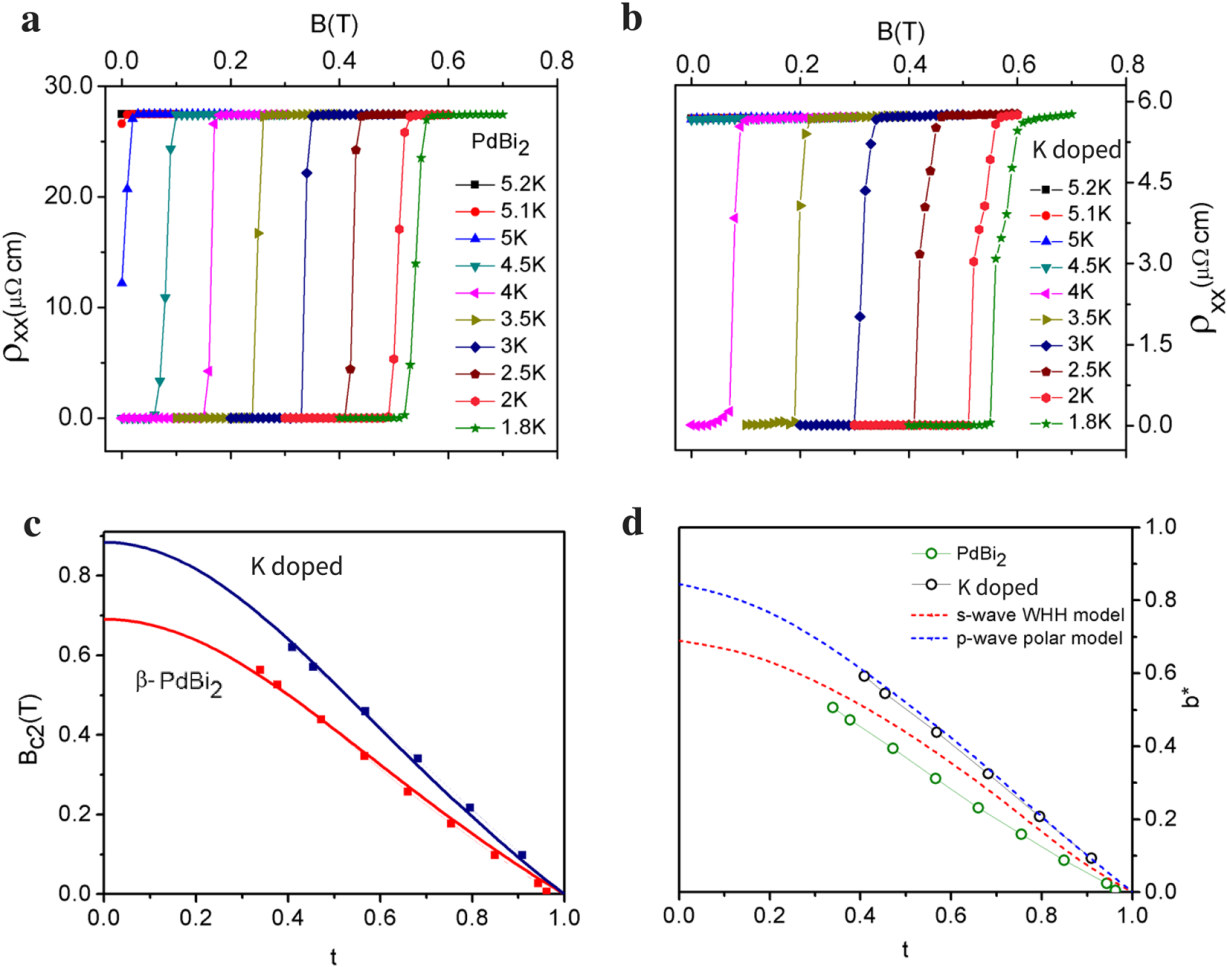
Upper-critical field analysis. (**a**,**b**), Variation of the upper critical field *B*_*c*2_ as a function of temperature in pristine and K-doped *β*-PdBi_2_. (**c**) The fit to *B*_*c*2_(*t*) = *B*_*c*2_(0)(1 − *t*^2^)/(1 + *t*^2^) is shown in red and blue for pristine and K-doped *β*-PdBi_2_ respectively. (**d**) Plot of the reduced upper critical field, b^*^ = *B*_*c*2_/|*dB*_*c*2_/*dt*|_*t*=1_ as a function of the reduced temperature *t* = *T*/*T*_*c*_. The red dash is the upper-limit for s-wave superconductivity according to the WHH model. A conventional superconductor with finite SOC and Maki parameter will be below the universal WHH model curve. K-doped *β*-PdBi_2_ lies above the upper-limit of WHH model and closer to the polar p-wave model, pointing to K-doped *β*-PdBi_2_ as an odd-parity superconductor.

To investigate the transport properties of the bulk superconductivity in more details, we study the upper-critical field limiting effect. In Fig. 3, the upper-critical field *B*_*c*2_ at different temperatures below *T*_*c*_ is plotted and extrapolated to T = 0 by using the form, *B*_*c*2_(*t*) = *B*_*c*2_(0)(1 − *t*^2^)/(1 + *t*^2^), where *t* = *T*/*T*_*c*_. *B*_*c*2_(0) is 0.69T for the pristine *β*-PdBi_2_ and a higher value of 0.89T is obtained for K-doped crystal in spite of it’s lower *T*_*c*_. Backscattering by impurities, even non-magnetic ones, suppresses odd-parity superconductivity as Anderson theorem does not hold^29,30^. Thus, we first check if the mean free path *l* is greater than the coherent length *ξ*_*c*2_. Using ${B}_{c2}={{\rm{\Phi }}}_{0}/2\pi {\xi }_{c2}^{2}$, where *ξ*_*c*2_ is the Ginzburg-Landau coherence length and Φ_0_ is the flux quantum, we obtain *ξ* = 19 nm for K-doped *β*-PdBi_2_ and *ξ* = 21 nm for pristine *β*-PdBi_2_. Assuming a spherical Fermi surface for simplicity, we have wavenumber *k*_*F*_ = (3*π*^2^*n*)^1/3^. Using n = 4.81 × 10^27^ m^−3^ derived from the linear part of *ρ*_*xy*_ (see^25^) for K-doped *β*-PdBi_2_ and the residual resitivity *ρ*_*o*_ from the longitudinal resistivity *ρ*_*xx*_, the the mean free path $l=\hslash {k}_{F}/{\rho }_{o}n{e}^{2}$ can be estimated. We find *l* = 75 nm. This *l*  >>  *ξ* combines contribution from both the surface and bulk state. If we use the only bulk carrier density, n = 3.4 × 10^28^ m^−3 25^, we have *l* = 22 nm, which is still greater than *ξ* = 19 nm. The doped crystal is sufficiently pure for odd-parity superconductivity. In contrast, we find that for the pristine sample, *l* = 8 nm < *ξ*.

Under the BCS theory, superconductivity can be limited by the orbital and spin effect of an external magnetic field. The orbital depairing effect is described by the WHH theory while the spin limiting effect is described by the Pauli paramagnetism formalism by equating the paramagnetic polarization energy to the SC condensation energy ${\chi }_{n}{({B}_{c2}^{p})}^{2}=N\mathrm{[0]}{{\rm{\Delta }}}^{2}$, where N[0] is the density of state, Δ is the SC gap, and from which the polarization field, ${B}_{c2}^{p}\mathrm{(0)}$ = 1.86 T_*c*_, is obtained. Under WHH theory in the clean limit, ${B}_{c2}^{orb}\mathrm{(0)}=0.72{T}_{c}|d{B}_{c2}/dT{|}_{{T}_{c}}$ = 0.75T for the doped sample. This is below the experimental *B*_*c*2_ value, suggesting that superconductivity is not orbital-limited.

The spin limiting effect is described by the Pauli paramagnetism, ${B}_{c2}^{p}\mathrm{(0)}$ = 1.86 T_*c*_ = 8.184T, which is way above the experimental B_*c*2_. So we have the relation: ${B}_{c2}^{orb}\mathrm{(0)}\, < \,{B}_{c2}\mathrm{(0)}\,\ll \,{B}_{c2}^{p}\mathrm{(0)}$, a relation which is also observed in Cu_*x*_Bi_2_Se_3_^16^. Now, when both the orbital and Pauli limiting effects are present, then ${B}_{c2}={B}_{c2}^{orb}\mathrm{(0)}/\sqrt{1+{\alpha }^{2}}$ = 0.74T. *α* here is the Maki parameter^31^; *α* = $\sqrt{2}{B}_{c2}^{orb}\mathrm{(0)/}{B}_{c2}^{p}\mathrm{(0)}$ = 0.13. The expected theoretical *B*_*c*2_ in the presence of both the orbital and spin limiting effects is lower than the experimental value 0.89T. We can possibly conclude that the Pauli limiting effect is also absent.

We can gain more insight by comparing the *B*_*c*2_(*T*) data with the well known theoretical model for s-wave^32^ and polar p-wave^33^. Figure 3d is the plot of the reduced upper critical field, b^*^ = *B*_*c*2_/|*dB*_*c*2_/*dt*|_*t*=1_ versus the reduced temperature *t* = *T*/*T*_*c*_ compared to the theoretical models for s-wave and polar p-wave SCs. For the doped crystal, we note that the experimental data exceeds the universal curve (upper-limit) for s-wave WHH model and fits better to the p-wave. The pristine *β*-PdBi_2_ in comparison lies below the upper-limit of the s-wave WHH theoretical prediction, as expected. It is important to note that WHH model presented in Fig. 3d is the universal curve (upper-limit), therefore the b^*^ VS *t* experimental data for s-wave superconductors does not have to fit the WHH model, it only has to be below the limit. The universal curve (upper-limit) is derived for *α* = *λ*_*so*_ = 0, where *α* and *λ*_*so*_ are the Maki parameter^31^ and the spin-orbit strength, respectively. *Non-zero α* and *λ*_*so*_ moves experimental b^*^ VS *t* curve below the theoretical universal curve. With non-zero *α* = $\mathrm{0.53|}d{B}_{c2}/dT{|}_{{T}_{c}}$ = 0.11 and finite *λ*_*so*_^34^ in *β*-PdBi_2_, the pristine crystal can be well described by the WHH model. This is in contrast to the doped crystal where b^*^ lies above the s-wave WHH upper-limit.

These commonly available transport experiments reveal unconventional superconducting properties in doped *β*-PdBi_2_, which are a departure from the conventional BCS theory. More direct experimental methods, Nuclear Magnetic Resonance (NMR) spectroscopy for example^35^, will provide more direct evidence for spin-triplet superconductivity. Once the bulk of our system is topologically nontrivial, bulk-boundary correspondence demands the emergence of topologically protected surface states, which are 2D helical Majorana surface fluid for 3D TRI TSC^7,8,36^. In the next section, we study the surface states in the superconducting state using Andreev spectroscopy. We briefly note that 2D Majorana surface of a 3D TSC is *distinct* from the 0D MZM edge state derived from a 1D TSC or from the vortex core of 2D TSC and exhibits distinct transport properties^8^.

## Surface Transport Properties

### Andreev spectroscopy

**Figure 4:**
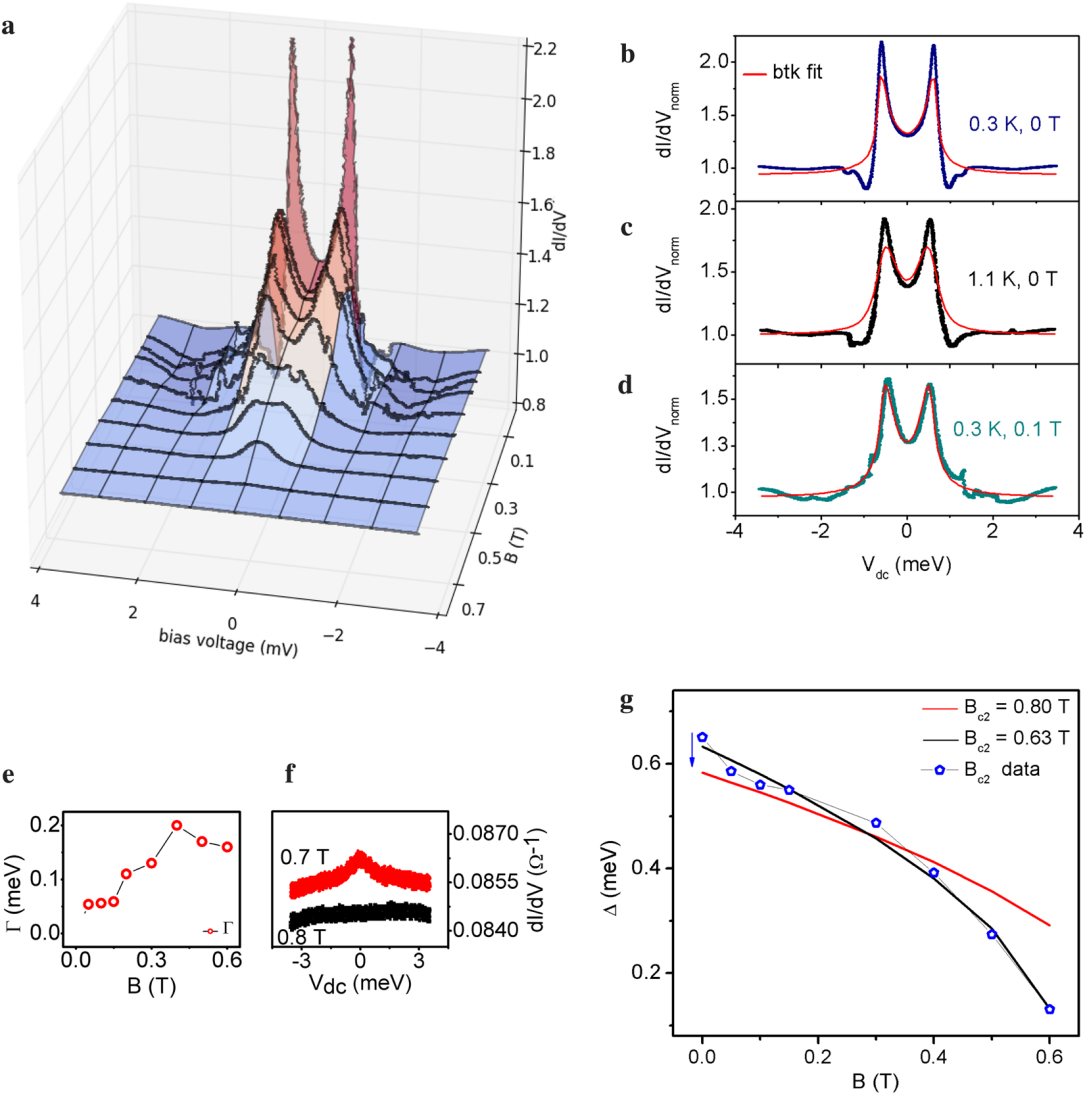
Point-contact spectroscopy. (**a**) Magnetic field dependence of the d*I*/d*V* vs bias voltage for K-doped *β*-PdBi_2_ at 0.3 K. (**b**,**c**) BTK fitting of the d*I*/d*V* spectrum at 0T for 0.3 K and 1.1 K. The fit is poor. In comparison, the fit is good at 0.1T in (**d**). (**e**) Field evolution of the quasi-particle lifetime broadening parameter Γ. (**f**) Close up view of d*I*/d*V* vs bias voltage at 0.7T and 0.8T. (**g**) Attempts to fit the gap with the BCS magnetic field dependence equation.

We performed ‘soft’ point-contact spectroscopy^37^ (see Supplementary Information S1 and S2) on K-doped *β*-PdBi_2_ cooled down to 300 mK, studying the magnetic field and temperature dependence of the differential conductance, d*I*/d*V*. In point-contact spectroscopy, z is representative of the barrier strength: z = 0 is Andreev spectroscopy; while z = ∞ (z ~ 5 in experiments) is tunneling spectroscopy. Here z = 0.4. We present the magnetic field dependence of d*I*/d*V* with current along the *ab* plane and the magnetic field along the *c* axis in Fig. 4.

We note that the spectrum at zero magnetic field is unconventional and looks remarkably different from the rest. In particular, in Fig. 4a (where we have normalized d*I*/d*V* to 1 as V → ∞), we see conductance *dips* at ±1 meV and conductance peaks exceeding the value predicted by the Blonder-Tinkham-Klapwijk (BTK) formalism of Andreev reflection^38^ for a conventional SC-insulator-normal metal interface (with z = 0.4). In fact, the peaks at 0.3 K exceed the theoretical maximum value of 2 (required by the Andreev process) for a gapped superconductor— indicative of the absence of conventional Andreev process— and thus might be a signature of the presence gapless surface states spanning the topological bulk gap.

To rule out trivial effects, let us recall the known causes of conductance dip in PCS d*I*/d*V* spectra. (i) *Critical current or heating effect*— dips at positions larger than the superconducting energy gap are often found in the spectrum when the contacts on the sample are in the thermal limit^39^. At the superconducting critical current, the superconductor turns into a normal metal, and when measurements are carried out in the thermal limit, the resistance of the bulk sample is measured in the d*I*/d*V* spectrum. Since the critical current required to limit superconductivity reduces with increasing magnetic field and temperature, these dips are found to occur at positions of decreasing lower bias voltage. In our experiments, the dip position does not reduce with increase in temperature and magnetic field (in fact it was only observed at zero magnetic field), so the critical current effect is ruled out (See Supplementary Information S2). (ii) *1D, 2D and 3D TSC* — topological superconductors feature dips at ±Δ. A simple physical explanation for this is the transfer of spectra weight from the states near the gap to make up for the in-gap states. In addition to the dips, 1D and 2D TSCs feature zero-bias conductance peak (ZBCP) due to Andreev bound state (ABS), while 3D TRI TSCs do not^8,10,14,40,41^.

For a finite potential barrier between the contact and an ideal 3D topological superconductor, dI/dV ∝ surface density of states, and differential conductance spectrum should produce a double peak structure^8,14,41^ — that is, ZBCP is *not* expected for fully gapped 3D TRI topological superconductors. It should be pointing out, however, that the tunneling conductance can feature a ZBCP due to various effects. In studies for superconducting 3D TI, remnant Dirac fermions from the normal state are found to modify in the superconducting state in two ways: one, it enhances the pair potential resulting in a larger gap for the surface superconductivity^42^. Two, if the Dirac surface states are well separated from the bulk, it can twist the surface Majorana cone, resulting in the ZBCP^41^. Furthermore, if the bulk superconductivity is not fully-gapped as is the case for Cu_*x*_Bi_2_Se_3_, for example, the tunneling conductance features a ZBCP^14^. Otherwise, in the ideal case, the differential conductance features a double peak.

Although the exact surface conductance spectrum of topological *β*-PdBi_2_, which will take into account the peculiar microscopics of the odd-parity bulk pairing allowed by the irreducible representations of its tetragonal *C*_4_ symmetry, has not been theoretically calculated yet; here, the presence of unconventional double conductance peak and nontrivial conductance dips at zero magnetic field are in good agreement with the general theoretical prediction for spin-triplet p-wave pairing for Balian-Werthamer (BW) phase of superfluid He-3 (see Fig. 4 in Yamakage *et al*.^41^). 3D TRI TSCs are the electronic analogue of He-3 BW phase. Alternately, we also consider the possibility that the unconventional surface conductance spectrum is a signature of surface *helical superconductivity* resulting from the Cooper pairing of the singly spin degenerate surface states present in the normal state. Such surface helical superconductivity are not s-waves but p-waves in nature and are considered as 2D topological superconductivity. This will be an intrinsic version of the surface helical superconductivity induced in heterostructures of s-wave superconductor and topological insulators^43^

Looking beyond the presence of unconventional surface states, we study its response to a time-reversal breaking perturbation, that is, a magnetic field. Analogous to the time-reversed Dirac fermions on the surface of a TI which are protected from backscattering, the superconducting state is expected host helical pairs of Majorana fermions which are robust against non-magnetic disturbances^36,43^. This physics is captured here: applying a magnetic field breaks time-reversal symmetry— and protection from scattering is lifted. The helical surface states are localized, the surface states are gapped, and the underlying gapped superconductivity described by the usual BTK-like spectrum is uncovered. We see in Fig. 4b–d that the spectrum under 0.1T at 0.3 K fits the BTK model for conventional superconductivity^38,44^ while that under zero magnetic field does not.

Next, we extracted the superconducting gap by fitting the experimental data at different magnetic fields to the BTK equation and attempted to fit its evolution with the prediction for a conventional Bardeen-Cooper-Schrieffer (BCS) superconductor: Δ(*B*) = Δ_0_(1 − *B*/*B*_*c*2_)^1/2^. Experimentally, *B*_*c*2_ is found to be somewhere between 0.7 and 0.8T according to the B field dependence of Andreev reflection as shown in Fig. 4f. However, the gap could not be described by BCS using 0.7T < *B*_*c*2_ < 0.8T; the misfit for 0.8T is shown in 4g. By making both the Δ_0_ and *B*_*c*2_ free parameters, we got the best fit with 0.63T. Clearly, as shown in Fig. 4f the crystal is still superconducting up till at least 0.7T. This proposes that the superconducting state might not be entirely described by conventional BCS theory.

## Discussion

**Figure 5:**
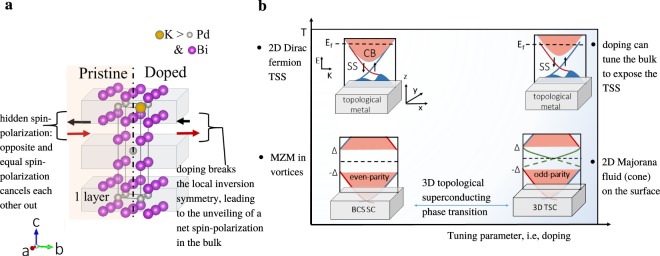
3D topological superconducting phase transition. (**a**) Centrosymmetric, layered materials can possess hidden spin-polarization in the bulk^46^; ‘hidden’ because the net spin-polarization cancels each other out. Naturally, the surfaces are spin-polarized, as is the case for pristine *β*-PdBi_2_ which possess trivial spin-polarized surface-states in addition to the topological surface-states. We proposed here that a net spin-polarization in the bulk can arise due to the doping effect which breaks the local inversion symmetry at random, intermittent sites. (**b**) Upper-left: topological metal: Fermi level E_*f*_ is placed above (or below) the topological surface states (SS). Upper-right: Doping depletes the bulk bands, exposing the SS. Lower-left: even-parity superconductivity in the bulk of a topological metal gaps out the bulk states and the Dirac SS; the superconductivity induced in the singly degenerate surface states resulting in a surface helical superconductivity, which is a 2D topological superconductor. In thin-films or cleaved materials, Majorana zero mode (MZM) (also referred to as Majorana bound state) can be trapped at the ends of the vortex cores. Lower-right: odd-parity bulk superconducting paring turns the bulk system into a 3D topological superconductor, which hosts in-gap, helical Majorana fermion surface states. *This is the subject of this paper*. While signatures of Majorana zero mode (MZM) have been demonstrated in several experiments, the 2D Majorana surface states unique to 3D TSC has been elusive. We propose that inducing a net spin-polarization in the bulk of centrosymmetric, layered topological metals can be a route to tunable 3D/bulk topological superconductivity in these materials.

In this paper, we have shown that in K-doped *β*-PdBi_2_ the bulk superconductivity is unconventional— a necessary condition for 3D topological superconductivity, and that the surface states are helical, a signature of this phase. We now address the question of *why* the doped system behaves so differently than the undoped one. We recall that sufficient conditions for topological superconductivity in a 3D TRI material are that the normal state Fermi surfaces enclose an odd number of TRIM *and* the fully-gapped bulk superconductivity pairing be odd under inversion. In pristine *β*-PdBi_2_ only the former condition met. Bulk superconductivity is s-wave^19,20,45^, so bulk topological superconductivity is not expected; instead, in a 2D thin-film one might get a Fu-Kane-like superconductor with edge Majorana zero modes in vortex cores^11,13^ (see lower-left corner of Fig. 5b), which are distinct from 2D helical Majorana surface states (lower-right corner of Fig. 5b).

Appropriate dopants can introduce Coulomb interaction, and in a layered centrosymmetric material, spin polarization^46,47^. Introducing either of these in a superconducting topological metal (which can host both s-wave and odd-parity because of intrinsic SOC), will suppress the s-wave pairing channel in favor of an odd-parity channel^21–23^. Here, on doping with K, we find that the c-lattice parameter increases^25^, indicating that the K ^+^ ions have replaced the smaller ions in *β*-PdBi_2_. This would lead to local inversion symmetry breaking without breaking the centrosymmetry of the bulk crystal, which is enough to unveil the hidden spin polarizations in the bulk of layered, centrosymmetric systems^46,47^. See Fig. 5a.

In the centrosymmetric Bi_2_Se_3_, superconductivity is induced by intercalating A = Cu, Nb, Sr into the non-superconducting parent compound. Recent transport studies on A_*x*_Bi_2_Se_3_ have shown the evidence of 2-fold pairing symmetry consistent with odd-parity, nematic superconductivity as opposed to the 6-fold symmetry of the hexagonal Bi_2_Se_3_ structure^35,48–50^. However, undoped Bi_2_Se_3_ does not display superconductivity at ambient pressures, so a topological-to-trivial superconductor phase transition does not occur in this system. The *fully-gapped* bulk superconductivity intrinsic to *β*-PdBi_2_ opens the possibility of an unprecedented topological phase transition in the superconducting state from a trivial to a topological superconductor. In case the topological superconductor first transitions into a magnetic phase, the accompanying critical point will present a unique and robust laboratory realization of emergent supersymmetry^24^.

In conclusion, we have presented the transport evidence for an *intrinsic* time-reversal-invariant topological superconductor in three dimensions. In the superconducting state, transport experiments give evidence that there is a different superconducting mechanism involved in the K-doped system compared to the pristine system. The upper-critical field experiments on K-doped *β*-PdBi_2_, in contrast to the pristine *β*-PdBi_2_, shows that the superconductivity exceeds WHH upper-limit for s-wave superconductivity; a signature of spin-triplet superconductivity. This is the sufficient condition for bulk topological superconductivity given that the normal state Fermi surface of *β*-PdBi_2_ encloses an odd number of single time-reversal invariant momenta (TRIM). Furthermore, Andreev spectroscopy reveals an unconventional spectrum at zero magnetic field, which possibly reflects the helical nature of 2D Majorana surface fluid, a surface manifestation of the non-trivial topology of the bulk. Different experimental approaches, in addition to material-specific theoretical studies, are required to determine the microscopics of the superconductivity. The coexistence of intrinsic superconductivity, topologically non-trivial bulk bands, and topological surface states in *β*-PdBi_2_ presents a unique material platform to study the interplay of Dirac fermions and Majorana fermions quasiparticles in condensed matter settings.

## Experimental Methods

### Transport measurements

The four-probe technique was used for the longitudinal resistance, *R*_*xx*_, and the Hall resistance, *R*_*xy*_, was acquired by the standard method. The magneto-resistance and magnetization measurements were carried out using Quantum Design Inc.’s Physical Properties Measurement System (PPMS. The magnetic properties of the samples were characterized using Quantum Design Inc.’s Magnetic Property Measurement System (MPMS). The device is able to detect small signals (≤10^−8^ emu) with great accuracy using the superconducting quantum interference device (SQUID) magnetometry technology. The MPMS can access temperatures as low as 1.8 K and can ramp the magnetic field up to 7T. The ‘soft’ point-contact spectroscopy was performed in an He-3 refrigerator. The current is past through a thin Au wire to the sample through a 30 *μ*m tiny drop of Ag nano-particle epoxy paint. To acquire the d*I*/d*V* data, a small AC current is superimposed with a sweeping DC current.

## Supplementary information

SUPPLEMENTARY MATERIALS: Possible transport evidence for three-dimensional topological superconductivity in doped β-PdBi<Subscript>2</Subscript>
