# Supplementary material for: Possible transport evidence for three-dimensional topological superconductivity in doped β-PdBi2
Source: Sci Rep. 2019 Aug 29;9:12504. doi: 10.1038/s41598-019-48906-7 (PMC6715648; doi:10.1038/s41598-019-48906-7)
Supplement: Supplementary file 1 — SUPPLEMENTARY MATERIALS: Possible transport evidence for three-dimensional topological superconductivity in doped β-PdBi2 [file 41598_2019_48906_MOESM1_ESM.pdf]

SUPPLEMENTARY MATERIALS: Possible transport evidence for three-dimensional topological superconductivity in doped β-$PdBi_2$

Ayo Kolapo * [1], Tingxin Li[2], Pavan Hosur[1], and John H. Miller[1]

[1]Texas Center for Superconductivity and Department of Physics, University of Houston, 3201 Cullen Boulevard, Houston, Texas 77204, USA

[2]Department of Physics and Astronomy, Rice University, Houston, Texas 77251, USA

July 10, 2019

*email: aykolapo@uh.edu

S1. ‘Soft’ Point-contact Spectroscopy Experiment

The point-contact spectroscopy set up is shown in Fig. S1. Ballistic transport is achieved through several channels of nanometer-sized silver particles contained in the silver epoxy [37, 14]. This method is in contrast to the hard metallic etched tip used in scanning tunneling spectroscopy (STS), hence, the ‘soft’ appellation. The dI/dV measurements were obtained by superimposing a constant 5 μA AC current with a sweeping DC current. The corresponding AC voltage can be picked up with great accuracy by the lock-in amplifier. The experiments were then carried out in a He-3 refrigerator.

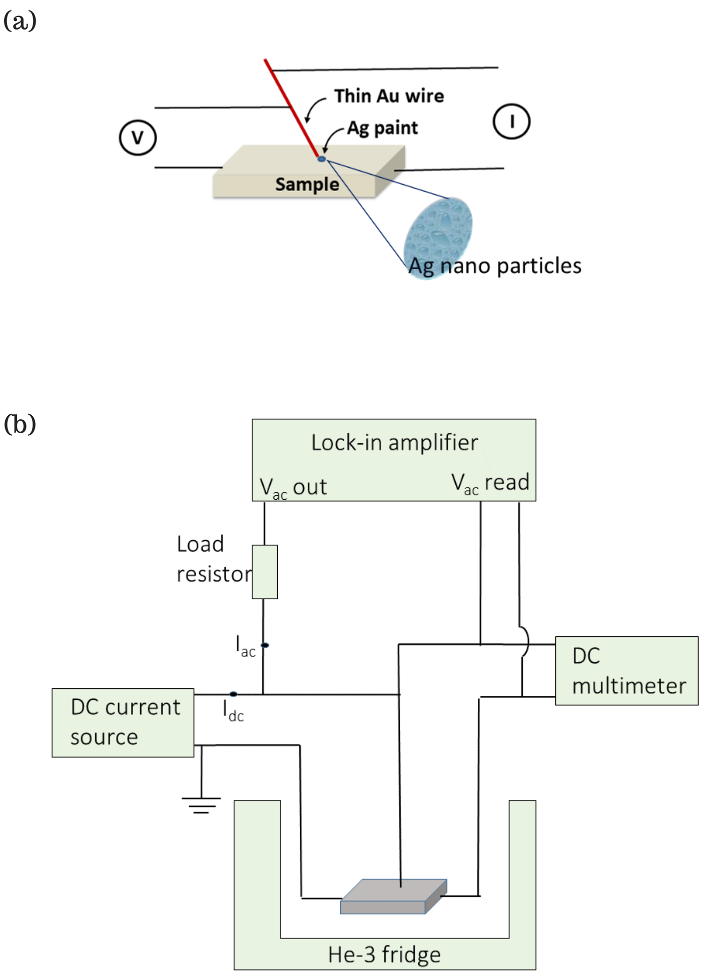

FIG. S1: (a) ’soft’ point-contact spectroscopy set up. The current is past through the thin Au wire to the sample through a 30 μm tiny drop of Ag nano-particle epoxy paint. (b) to acquire the dI/dV data, a small AC current is superimposed with a sweeping DC current. It shown in the text that the corresponding AC voltage is proportional to the differential conductance.

S2. Andreev Spectroscopy

First, we check if the 'soft' point-contact spectroscopy experiment is in the ballistic or intermediate regime where spectroscopy information can be observed. When the mean free path l is greater than contact radius a, $l \gg a$, the electrons can accelerate ballistically without losing kinetic energy due to scattering [39, 37]. In the opposite case, the diffusive regime, $a \gg l$, energy is lost due to inelastic scattering at the point contact interface. In a 'soft' point-contact spectroscopy experiment where the contact is achieved through several channels, a can be indirectly obtained from

$$a = \sqrt{\frac{4\rho l}{3\pi R_N}} \tag{1}$$

where $R_N \approx R_s$ = Sharvin resistance

There are two things to note: (i) since there are many channels, a derived might be larger than the actual contact size through which the transport occurs, and (ii) using the equation given by the Sharvin resistance, we assume the point contact setup is in the ballistic regime.

We find that $a = 12$ nm. $l = 75$ nm is obtained in Ref. [25]. $l \gg a$, our point-contact spectroscopy experiment is in the ballistic regime.

In Fig. S2, we comment and highlight on the feature in the dI/dV spectrum presented in the main text. The BTK formalism for the Andreev reflection can be written as:

$$\frac{dI}{dV}_{Norm} \propto \int_{-\infty}^{\infty} dE \frac{df(E+eV)}{df(eV)} [1 + A(E) - B(E)] \tag{2}$$

where $\frac{dI}{dV}_{Norm} = \frac{\frac{dI}{dV}_{NS}}{\frac{dI}{dV}_{N}}$, and $A(E)$ is the probability amplitude for Andreev reflection, and $B(E)$ is the probability amplitude for specular reflection. At $T = 0$ and in the limit z $\rightarrow 0$, then for a particle with $E < \Delta$ undergoing a complete Andreev reflection, we have $\frac{dI}{dV} = 2$, because B(E) $= 0$. When T and $B(E)$ are finite, $\frac{dI}{dV} < 2$.

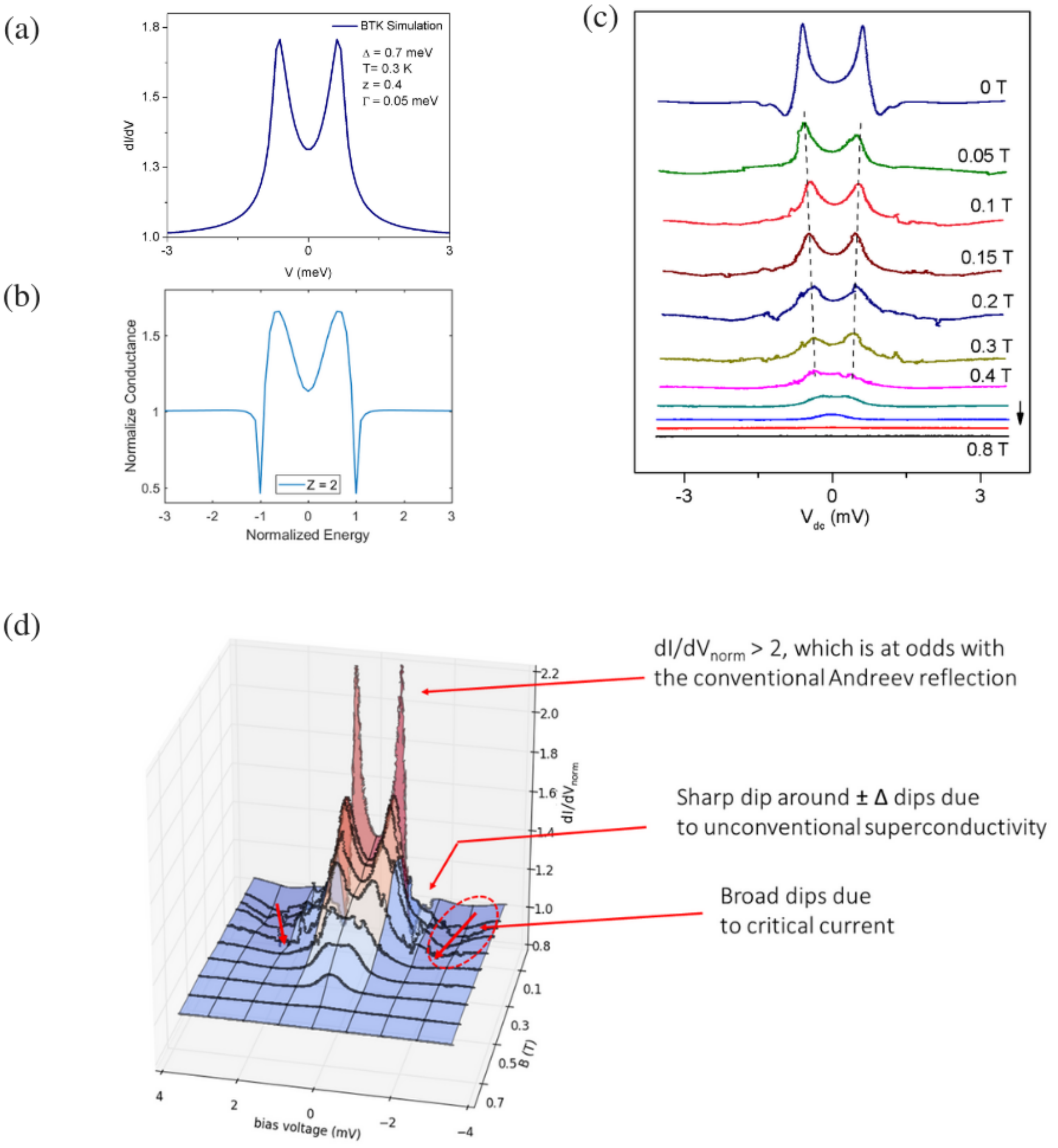

FIG. S2: (a) the simulation for the following parameter, similar to actual experimental values: superconducting gap, $\Delta = 0.7$ meV, barrier strength, z $= 0.4$, temperature, T $=$ 0.3 K, and quasiparticle interference parameter $\Gamma = 0.05$ meV (b) Simulation of the spin-triplet p-wave pairing for Balian-Werthamer (BW) phase of superfluid He-3 (c) $\mathrm{d}I/\mathrm{d}V$ spectrum at 300 mK. The plots have been shifted for clarity. (d) 3D plot of the $\mathrm{d}I/\mathrm{d}V$ spectrum depicting unconventional conductance dip and the dramatic increase of $\mathrm{d}I/\mathrm{d}V$ at zero magnetic field.

In this study, the spectroscopy is consistent with the BTK formalism when the magnetic field is turned ON (that is, when the magnetic field localizes the helical surface states). In the absence of the magnetic, we see the normalized differential conductance suddenly rising to > 2. We emphasise here the sudden rise of the conductance.

More revealing is the symmetric conductance dip with only occurs at zero mag-

netic field, independent of temperature. The conductance peak around $\pm\Delta$ is shown not to be due to critical current. This is because the bias voltages at which the conductance dips due to critical currents occur are expected to *decrease* with an increasing magnetic field or temperature limiting the superconducting gap (as illustrated in the FIG. S2(d)). The dips at zero field do not follow these physics. 3D/bulk time-reversal invariant topological superconductivity is the "superconducting" analogue of the "superfluid" He-3 BW phase. They both are expected to host Majorana cone surface states. The surface spectra of He-3 BW phase is well studied: the distinguishing feature is the double conductance dip (See Fig 2S(b)). Although for a superconductor, the spectra might be complicated by the details of the Fermi surfaces; nonetheless, the double conductance dip observed in K-doped β-$PdBi_2$ is consistent with the qualitative prediction. For example, the same features—double conductance peaks and double conductance dips features—are qualitatively present in the fully-gapped, odd-parity, spin-triplet pairing (so-called Δ_2) of Cu-doped Bi_2Se_3 [See supplementary information in 14].

In this paper, we have shown that K-doped β-$PdBi_2$ possibly satisfies the necessary condition for bulk topological superconductivity, therefore helical surface states is guaranteed to exist (whether it's detected in transport measurements or not). This suggests that the anomalous zero magnetic field Andreev spectrum might be signatures of helical in-gap states, which in this situation are expected to be 2D surface Majorana fluids (distinct from the 0D Majorana zero mode that has been detected in nanowire heterostructures and in vortex cores of 2D topological superconductors).

Perhaps, one of the 'smoking gun' feature of Majorana surface states in transport experiment is that, in contrast to the 2D transport of electrons in a disordered system which are susceptible to quantum interference, 2D Majorana fluid are immune to nonmagnetic perturbation [9]. Here, taking a closer look at the data, we found that the root-mean-squared (rms) of the amplitude of oscillations, rms δG, of the conductance spectrum in the absence of a magnetic field is *6 times smaller* than when the magnetic field is turned on. This interesting observation will be studied in details in further experiments.
